# “The wheel is come full circle” – Sustainability in surgery

**DOI:** 10.1016/j.amsu.2022.103473

**Published:** 2022-03-05

**Authors:** Qurrat Al Ain Atif

**Affiliations:** General Surgery, Darent Valley Hospital, Dartford and Gravesham NHS Trust, Dartford, UK

Climate change, global warming and carbon footprint are very powerful and overwhelming terms which have stirred global unrest for the past decade. Global efforts are on way to counter these effects and make sustainable changes that are eco-friendly.^1^.

Healthcare sector, a major contributor to the carbon footprint (5%) 2, has also mobilised resources into identifying and dealing with areas that might be causing environmental harm. There is an uproar of “sustainability in surgery” and leaders and representatives have proposed ideas and pathways to support that notion.

Among all the change and adaptation, one seems to wonder as to how we reached this stage. While revisiting “reduce, reuse and recycle” adage, we need to point to the fact that surgery was in fact, historically, far more sustainable. Some three decades ago, reusable linen, reusable medical and surgical equipment, limited patient inflow and lesser number of surgeries were the trends. The sterilization techniques were not standardized 3 which would have caused higher risk of infections but instead of focusing on that surgery took an unusual turn.

1990s saw a paradigm shift in surgical culture. Healthcare professionals followed the myths of reduction in infection risk secondary to using disposable items.^3^ This brings us to medical equipment industry, capitalism and profit seeking companies. The label of modernising and advancing healthcare was so catchy that all else was forgotten. We started relying on single-use medical products without bearing in mind the consequences that would follow. Every product in separate plastic packaging, adding to the landfill. Not to mention the additional costs to the healthcare for every single-use product utilised in terms of production, procurement, transportation and disposal. On the other hand, a systematic review found out that expensive equipment like endoscopes have a much higher decontamination, maintenance and repair cost as compared to single-use scopes.^4^ One might then argue about the impact of sterilization on finances as well as the environment. However, a recent study showed that if surgical equipment is decontaminated and packaged as sets, it reduces carbon footprint as well as financial costs, significantly.^5^ Understandably though, this might not be achievable for every piece of equipment.

With the realisation of the harm being caused, we now are focused to reduce if not reverse the damage. Taking a U-turn and heading back to reusability. More than recycling, we need to focus on reduce and reuse to make an impact. For example, we need to limit the number of instruments utilised in a surgery, promoting reusable products like surgical gowns and drapes, minimising packaging. We need to find the fine balance between patient safety, cost effectiveness and environmental impact. The ultimate goal being uncompromised patient care with financial, social and environmental sustainability in mind.

In summary, we have come from switching from reusable to single-use and back to advocating reusable medical products within a matter of a few decades. Or in the words of William Shakespeare “the wheel is come full circle”.

## Provenance and peer review

Not commissioned, externally peer reviewed.

## Ethical approval

None required.

## Sources of funding

None.

## Consent

NAD.

## Author contribution

Dr Qurrat Al Ain Atif did literature search and manuscript writing as well as proof reading.

## Registration of research studies

1. Name of the registry:

2. Unique Identifying number or registration ID:

3. Hyperlink to your specific registration (must be publicly accessible and will be checked):

## Conflicts of interest

None.

## Guarantor

Corresponding author is guarantor of submission.

## References

[1] Pradere B., Mallet R., de La Taille A., Bladou F., Prunet D., Beurrier S. (2022). Climate-smart actions in the operating theatre for improving sustainability practices: a systematic review. Eur. Urol..

[2] Lenzen M., Malik A., Li M., Fry j, Weis H., Pichler P.P. (2020 Jul). The environmental footprint of healthcare: a global assessment. Lancet Planet Health.

[3] Bhutta M.F. (2021 Nov). Our over-reliance on single-use equipment in the operating theatre is misguided, irrational and harming our planet. Ann. R. Coll. Surg. Engl..

[4] Rizan C., Lillywhite R., Reed M., Bhutta M.F. (2022 Feb 1). Minimising carbon and financial costs of steam sterilisation and packaging of reusable surgical instruments. Br. J. Surg..

[5] Mouritsen J.M., Ehlers L., Kovaleva J., El-Boghdadly K. (2020 Apr). A systematic review and cost effectiveness analysis of reusable vs. single-use flexible bronchoscopes. Anaesthesia.

